# Impact of prenatal tobacco smoking on infant telomere length trajectory and ADHD symptoms at 18 months: a longitudinal cohort study

**DOI:** 10.1186/s12916-022-02340-1

**Published:** 2022-04-28

**Authors:** Meghan P. Howell, Christopher W. Jones, Cade A. Herman, Celia V. Mayne, Camilo Fernandez, Katherine P. Theall, Kyle C. Esteves, Stacy S. Drury

**Affiliations:** 1 Department of Pediatrics, Tulane University School of Medicine, 1430 Tulane Avenue, #8526, New Orleans, Louisiana 70112 USA; 2 Department of Psychiatry, Perelman School of Medicine, University of Pennsylvania, Philadelphia, PA USA; 3 Tufts University School of Medicine, Boston, MA USA; 4 Department of Psychiatry and Behavioral Science, Tulane University School of Medicine, 1430 Tulane Avenue, #8526, New Orleans, Louisiana 70112 USA; 5 Department of Orthopedic Surgery, Tulane University School of Medicine, 1430 Tulane Avenue, #8526, New Orleans, Louisiana 70112 USA; 6 Department of Epidemiology, Tulane University School of Public Health and Tropical Medicine, New Orleans, LA USA; 7 Global Community Health and Behavioral Sciences, Tulane University School of Public Health and Tropical Medicine, 1430 Tulane Avenue, #8526, New Orleans, Louisiana 70112 USA; 8 Clinical Neuroscience Research Center, Tulane University School of Medicine, 1430 Tulane Avenue, #8526, New Orleans, Louisiana 70112 USA

**Keywords:** Smoking, Tobacco, Prenatal, Telomere length, Telomere attrition, Maternal depression, Attention deficit/hyperactivity disorder

## Abstract

**Background:**

Prenatal maternal tobacco smoking is a predictor of child attention-deficit/hyperactivity disorder (ADHD) and is associated with offspring telomere length (TL). In this study, we examine the relationship between maternal prenatal smoking, infant TL, and maternal report of early childhood symptoms of ADHD.

**Methods:**

One-hundred and eighty-one mother-infant dyads were followed prospectively for the infant’s first 18 months of life. Prenatal smoking was assessed from maternal report and medical records. TL was measured from infant buccal swab DNA obtained across the first 18 months of life. ADHD symptoms were obtained from maternal report on the Child Behavior Check List. Multiple regression models tested the relation between prenatal smoking and both ADHD symptoms and infant TL. Additional analyses tested whether the change in infant TL influenced the relation between prenatal smoking and ADHD symptoms.

**Results:**

Sixteen percent of mothers reported prenatal smoking. Infant TL at 4, 12, and 18 months of age were correlated. Consistent with previous cross-sectional studies linking shorter offspring TL to maternal prenatal smoking, maternal prenatal smoking predicted greater telomere shortening from four to 18 months of infant age (*β* = − 5.797, 95% CI [-10.207, -1.386]; *p* = 0.010). Maternal depression was positively associated with both prenatal smoking (odds ratio (OR): 4.614, 95% CI [1.733, 12.282]; *p* = 0.002) and child ADHD symptoms (*β* = 4.713, 95% CI [2.073, 7.354]; *p* = 0.0006). To prevent confounding, analyses examined the relation between TL, ADHD symptoms, and prenatal smoking only in non-depressed mothers. In non-depressed mothers, infant TL attrition across the first 18 months moderated the relation between smoking and child ADHD.

**Conclusions:**

The findings extend previous studies linking prenatal smoking to shorter infant TL by providing data demonstrating the effect on TL trajectory. The relation between prenatal smoking and early infant ADHD symptoms was moderated by the change in TL. The findings provide novel initial evidence suggesting that TL dynamics are one mechanistic pathway influencing the relation between maternal prenatal smoking and ADHD.

**Supplementary Information:**

The online version contains supplementary material available at 10.1186/s12916-022-02340-1.

## Background

Despite the established negative effects of prenatal maternal smoking on her offspring, as of 2016, one in 14 mothers in the USA reports smoking during pregnancy [1]. Maternal prenatal smoking is associated with preterm birth [2], placental abruption [3], low birthweight and fetal growth restriction [2–4], stillbirth [3], and sudden infant death syndrome [3]. These adverse effects extend into childhood with links to childhood asthma [5] and obesity [6, 7], and meta-analytic data associates maternal prenatal smoking with attention deficit/hyperactivity disorder (ADHD) [8, 9]. The pathophysiological mechanisms of these broad consequences of prenatal maternal smoking are poorly understood; however, changes to mitochondrial function, oxidative stress, and placental apoptosis are hypothesized contributors [10–12]. One molecular marker tied to oxidative stress and cellular apoptosis that is also associated with smoking is telomere length (TL) [13].

Telomeres are the protective nucleoprotein cap at the end of each chromosome that, due to the end replication problem, shorten with each cell cycle [14]. Both oxidative stress and DNA damage accelerate telomere loss. In adults, shorter TL is associated with smoking [13], with some evidence of a dose dependent effect [15, 16]. A recent meta-analysis found shorter TL in smoking adults compared to non-smoking adults; however, no association was seen between smoking and the rate of TL attrition across the life span. The authors suggested that the observed cross-sectional relationship, in adults, between shorter TL and smoking was due to unmeasured effects early in life [17]. Beyond these direct effects within an individual, maternal prenatal smoking has been previously associated with shorter TL in her offspring [18–22].

To date, seven studies have reported on the relation between maternal prenatal smoking and TL in her offspring at different ages from different sources of DNA [20, 21, 23–27]. Of these studies, six found that exposure to prenatal smoke was associated with shorter TL in offspring [20, 21, 23–25, 27]; however, one study reported maternal smoking associated with longer newborn TL [26]. Shorter cord blood TL has also been reported in infants born to mothers exposed to second-hand smoke prenatally [21], as well as in mothers with higher concentrations of urinary cadmium—one of the main toxic components in cigarette smoke [27]. Maternal smoking during pregnancy has been associated with shorter cord blood TL [24]; however, maternal prenatal smoking was also reported to be associated with *longer* TL in lymphocyte subpopulations. In this study, the authors hypothesized that longer TL was the consequence of a shift in the replicative history of specific lymphocyte sub-populations [26]. Beyond effects on newborn TL, maternal prenatal smoking has also been associated with shorter TL in two cross-sectional studies in older children [20] and adolescents [25]. Collectively, these studies indicate both an initial and persistent impact of maternal prenatal smoking on offspring TL; however, the directionality and potential differences in the relation due to the tissue type in which TL was measured require further exploration. To date, no studies have explored the trajectory of offspring TL in relation to prenatal smoking, nor have any studies tested the association between prenatal smoking, TL, and child outcomes.

Accelerated TL shortening, in both adults and pediatric populations, has been related to many aging related diseases including cardiovascular disease [28], obesity [29], and diabetes [30]. Further accelerated TL loss has been associated with environmental exposures related to increased oxidative stress [31]. Lastly, TL in preclinical and animal models has been associated with cellular differentiation, cellular senescence, and cellular aging [32]. In addition to the established link between maternal prenatal smoking and ADHD in her offspring, studies have reported associations between ADHD and TL. Costa Dde et al. demonstrated that higher levels of hyperactivity-impulsivity were associated with shorter TL in both children and their mothers [33]. In contrast, Momany et al. demonstrated that previous childhood hyperactivity and impulsivity symptoms, but not current adult symptoms or persistence of ADHD symptoms, were associated with longer TL [34]. Dyskeratosis congenita is a telomeropathy with higher incidence of ADHD, suggesting a link between telomere pathology and ADHD. Given these results, additional prospective studies exploring the link between TL and ADHD, particularly given the evidence associating maternal smoking with both TL and ADHD, are needed.

This study examined the association between maternal prenatal smoking and infant TL trajectory across the first 18 months of life and how the change in TL influenced the relation between prenatal maternal smoking and maternal report of child ADHD symptoms at 18 months of age. First, we hypothesized that fetal exposure to maternal prenatal smoking would be associated with accelerated TL loss across the first 18 months of life in the infant. Second, we hypothesized that, consistent with previous studies, maternal prenatal smoking would be associated with higher ADHD symptoms in children at 18 months of age. Lastly, we hypothesized that the change in TL would moderate the association between maternal prenatal smoking and ADHD symptoms.

## Methods

### Sample

**Fig. 1:**
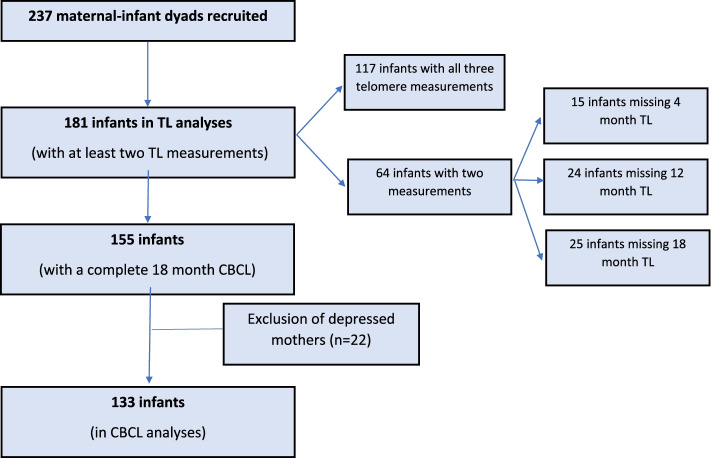
Flow chart of subject inclusion/exclusion criteria

A total of 237 mother-infant dyads were recruited from prenatal clinics, Women, Infants, and Children (WIC) clinics, and other ongoing University studies. Women were enrolled at any point during pregnancy and up to four months after delivery. Mothers were excluded if they were less than 18 years of age or non-English speakers. Mothers provided information about multiple levels of their and their infant’s social ecology, including demographic data such as race and educational attainment by using an interview-assisted computer survey administered face-to-face by a trained research assistant (Questionnaire Development System; Nova Research, Bethesda, Maryland). Oral responses were recorded onto an electronic tablet. Study visits with the infant and mother together were conducted when the infant was 4, 12, and 18 months of age. This study was approved by the University Institutional Review Board (Fig. 1).

### Measures

#### Maternal prenatal smoking

Maternal prenatal smoking was defined using three methods to capture smoking at any point during pregnancy: (1) statement of tobacco use during pregnancy in maternal prenatal medical records, (2) maternal report of prenatal smoking during the prenatal interview, or (3) maternal retrospective report of smoking at any time point during pregnancy at the infant’s 4-month visit. One endorsement through any of these approaches was considered positive for maternal prenatal smoking and this was used to generate a dichotomized prenatal exposure variable for analyses. Component prenatal smoking variables used to define the composite maternal prenatal smoking were positively correlated with correlation coefficients ranging from 0.61 to 0.83 (ps < 0.0001).

#### Socioeconomic status (SES) index

A SES index was created from a sum of maternal education, employment, home ownership, income, savings and government assistance status (range: 0–6). Education was scored “0” for less than high school and “1” for high school or greater. Employment was scored “0” for less than full time and “1” for full time. Home ownership was scored “0” for renting and “1” for ownership. Income was scored “0” for less than $24,999 annual income and “1” for greater than $25,000. Savings was scored “0” for less than $500 and “1” for $500 or greater. Government assistance was scored “0” for assistance and “1” for no assistance [35].

#### Pregnancy-related data

Pregnancy-related data and newborn outcomes were abstracted from medical records, which were defined as gestational age, infant birthweight, and clinical pregnancy complications. Gestational age ranged from 29 to 43 weeks. Clinical complications during pregnancy were also abstracted from medical records and included gestational hypertension, gestational diabetes, fetal growth restriction, and preeclampsia/eclampsia. Due to the low prevalence of individual pregnancy complications in this study, we generated a composite, dichotomous pregnancy complication variable where, if one or more complications were present, then the pregnancy was scored as “1,” and if none were present, the pregnancy was scored “0” [36].

#### Maternal depression

At 18 months of infant age, mothers reported on their current depressive symptoms with the Beck Depression Inventory-II [37]. A clinical threshold score of 14 or higher was classified as positive for maternal depression.

#### Attention-deficit/hyperactivity disorder (ADHD) symptoms

ADHD symptoms were obtained from maternal report on the Child Behavior Check List (CBCL) DSM-oriented attention deficit/hyperactivity problems subscale at 18 months of infant age [38]. The *T*-score was utilized in analyses.

#### Infant buccal telomere length (TL)

DNA was collected from infants using buccal swabs at 4, 12, and 18 months of age using Isohelix SK1 buccal swabs (Cell Projects, Kent, UK). Swabs were air dried and then stored in a sealed tube with a dessicator pellet at 4 °C until DNA was extracted. Genomic DNA was isolated from buccal swabs using the QIAamp DNA Mini Kit protocol (Qiagen, Valencia, CA; Invitrogen, Carlsbad, CA). All DNA samples were evaluated for double-stranded integrity and concentration by Qubit dsDNA BR assay kit (Invitrogen, Carlsbad, CA) and purity by NanoDrop-2000 (Thermo Fisher Scientific, Waltham, MA). Once extracted, DNA was stored at − 20 °C and underwent no more than three freeze thaw cycles. The average relative TL was determined from the telomere repeat copy number to single gene (albumin) copy number (T/S) ratio by using an adapted monochrome multiplex quantitative real-time polymerase chain reaction (PCR) and a BioRad CFX96 [39]. Longitudinal TL measurement was conducted as previously reported [40]. Briefly, all infant buccal DNA samples from the same infant were run on the same plates in triplicate with a 7-point standard curve (0.0313 ng to 2 ng) using a single pooled control buccal DNA sample for all plates. Each plate was run in duplicate with all samples in a different well position. Thus, six replicates of both the single copy gene and the telomere repeat were available for each individual at each time point. PCR efficiency criteria for telomere and albumin reactions were 90% to 110%. Coefficients of variations (CVs) were calculated within each triplicate (CV criteria < 10%) and between plates (CV criteria < 6%). Samples with unacceptably high CVs (> 10% intra- or > 6% interassay CV) were repeated. All samples were determined by the average of the triplicates from both plates. Additionally, all samples from each time point were required to pass DNA quality control metrics for any sample measurement to be included. Single measurement and average measurement intra class correlation coefficients (ICCs) were calculated for all TL measurement by each time point using R [41]. R code available on the Telomere Research Network web page (tulane.trn.edu).

### Statistical analysis

Descriptive, bivariable, and multivariable analyses were conducted with SAS version 9.4 (SAS Institute, Inc. Cary, NC). Descriptive statistics characterized participants overall, as well as between maternal smoking and nonsmoking groups. Infant TL at any time point did not differ from a normal distribution as judged by Q-Q plots. Correlation analyses examined associations between covariates and outcomes. Single imputation was performed for missing TL measures using the mean TL by infant sex and age at the missing TL time point; this was conducted only for infants with a completed CBCL form who were missing either a 4-month or 18-month TL measurement resulting in 4.2% of imputed TL measurements (*n* = 20 TL estimates). All statistical models were conducted with and without imputed TL data and did not differ substantively [42].

#### Maternal prenatal smoking and infant TL trajectory

Multilevel mixed-effects linear regression models (MLM) were executed using *PROC MIXED* in SAS to account for clustering of repeated TL measurements within an individual, and the *CL* option was used to produce 95% confidence limits (CL). An unstructured covariance matrix was used due to the repeated TL measurements within an individual, and it produced the best model fit (AIC = 847.6). The MLM included the individual-specific intercept as a random effect, and maternal prenatal smoking and other covariates were included as fixed effects. The MLMs examined the impact of maternal prenatal smoking on infant TL in the following steps: (1) the main effect of maternal prenatal smoking across infant TL time points accounting for only infant age (i.e., for linear trajectories in TL) and age^2^ (i.e., for nonlinear trajectories in TL), (2) an MLM to examine individual growth curves via the interaction of infant age and maternal prenatal smoking status, and (3) an adjusted individual growth curve model accounting for the following covariates: race, infant sex, birthweight, and SES. Models were run with and without maternal postnatal depression and did not significantly contribute to the models; therefore, it was not included as a covariate.

#### Maternal prenatal smoking and infant ADHD symptoms at 18-months of age

Linear regression tested the adjusted relation of maternal prenatal smoking and infant CBCL ADHD *T*-score. The adjusted model accounted for infant sex, race, SES, and current maternal depression (dichotomized). Correlation analyses between predictors revealed a significant risk of confound when including maternal depression due to significant and substantial relations with both the independent (i.e., maternal prenatal smoking) and dependent variables (i.e., ADHD *T*-score) (Fig. S1). Therefore, evaluation of the relation between prenatal smoking and early childhood ADHD symptoms was conducted in non-depressed mothers only (*n* = 133).

#### The moderation of maternal prenatal smoking and infant ADHD symptoms by TL attrition

To examine whether TL attrition moderated the association between maternal prenatal smoking and CBCL ADHD *T*-score, a change score was generated from the difference between infant 4-month TL and 18-month TL (i.e., ∆TL = 4-month TL–18-month TL). No association was observed between TL attrition and CBCL ADHD *T*-score, and thus, moderation was tested in the following step-wise fashion: (1) the independent effects of prenatal tobacco smoking and TL attrition on infant ADHD symptoms and (2) the independent effects of prenatal smoking and TL attrition, as well as their interaction on infant ADHD symptoms. The adjusted model accounted for infant sex, race, and SES; consistent with the analysis of maternal prenatal smoking on infant ADHD symptoms, the analysis was conducted only in non-depressed mothers (*n* = 133).

## Results

### Sample characteristics

Inclusion criterion for the study on the impact of maternal prenatal smoking on infant TL trajectory required a minimum of two infant TL measurements, which yielded four-hundred and seventy-nine infant TL measurements obtained from a total of *N* = 181 infants. Twenty TL estimates were imputed to facilitate moderation analyses and resulted in a final sample of *N* = 499 TL point estimates. Of the *N* = 181 infants included in the study, *n* = 117 infants had TL measurements from all three time points and *n* = 64 infants had measurements from two time points (Fig. 1). Of the *n* = 64 infants missing one TL measurement, *n* = 15 infants were missing TL at 4 months of age, *n* = 24 infants were missing TL at 12 months, and *n* = 25 infants were missing TL at 18 months. Missing TL measurements were either the result of missing one visit (*n* = 40) or difficulty with collection of the buccal sample from the infant and a resulting DNA concentration that was too low for analysis (*n* = 24). No samples were excluded due to failed DNA quality control or TL measurement quality control; however, approximately 5% of samples were repeated due to replicate inconsistencies of one time point. The overall CV was 0.0228. Single measurement and average measurement ICCs were excellent and ranged from 0.924 to 0.991 (Table 1).

**Table 1:** Intraclass correlation coefficients for test-retest reliability of T/S ratios at 4, 12, and 18 months

	Intraclass correlation coefficient (ICC)			
Time point	Model^**a**^	ICC value	95% confidence interval	***p***-value
4 months	ICC (A,1)	0.948	0.925–0.966	< 0.001
	ICC (A,k)	0.991	0.987–0.994	< 0.001
12 months	ICC (A,1)	0.944	0.916–0.965	< 0.001
	ICC (A,k)	0.99	0.985–0.994	< 0.001
18 months	ICC (A,1)	0.924	0.884–0.953	< 0.001
	ICC (A,k)	0.987	0.979–0.992	< 0.001

^a^Model structure, type, and definition based on the McGraw and Wong classification. All computations considered two-way random effect models, absolute agreement, for ICC (A,1), single measurement; and ICC (A,k), average measurements

Inclusion criteria for the study on the impact of maternal prenatal smoking on early childhood ADHD symptoms required a complete CBCL form at 18 months of age and a minimum of two infant TL measurements, yielding a final sample of *n* = 155 infants due to *n* = 27 infants without CBCL data. No differences in sample demographics were observed between the total *N* = 181 infants in the TL trajectory study and the *n* = 155 infants with CBCL data (Fig. 1). Furthermore, infants with imputed TL attrition data did not differ on outcomes relative to infants without TL attrition data including the prevalence of mothers who smoked prenatally (*p* = 0.54), sex (*X* = 0.03; *p* = 0.85), race (*p* = 0.84), maternal postnatal depression (*p* = 0.74), maternal age at conception (*t* = 0.92; *p* = 36), infant birthweight (*t* = 0.34; *p* = 0.74), SES (*t* = − 1.18; *p* = 0.24), or TL attrition (*t* = − 1.27; *p* = 0.21).

Characteristics of the sample are presented in Table 2, and correlations between study variables are presented in Table 3. Most mothers were recruited during the third trimester of pregnancy (*n* = 100; 55.3%) with 29.3% in the second (*n* = 53), 5.5% in the first (*n* = 10), and 9.9% postnatally (*n* = 18). Sixteen percent of mothers reported prenatal smoking (*n* = 29). Mothers who endorsed prenatal smoking reported lower SES (*ρ* = − 0.18; *p* = 0.015) and had lower birthweight infants (*ρ* = − 0.19; *p* = 0.018). No differences in prenatal smoking frequencies were observed by race; however, race differences in SES were observed (*F*(2178) = 28.14; *p* < 0.0001). White mothers reported higher SES than both Black mothers (*t* = − 7.46; *p* < 0.0001) and mothers of other race (*t* = 2.33; *p* = 0.022); Black mothers reported lower SES than mothers of other race (*t* = − 2.45; *p* = 0.016). Infant birthweight was positively associated with SES (*ρ* = 0.30; *p* < 0.0001) and varied by race, with Black mothers having lower birthweight infants than white or other race mothers (*F*(2178) = 13.03; *p* < 0.0001). Lower infant birthweight was associated with maternal pregnancy complications (*ρ* = − 0.22; *p* = 0.003). Infant TL was significantly correlated across time points (*ρ* = 0.24–0.25). White and other race infants exhibited shorter TL at 12 months of age relative to Black infants (*ρ* = − 0.21; *p* = 0.007). Prenatal smoking was associated with longer infant TL at 4 months (*ρ* = 0.16; *p* = 0.042) and shorter infant TL at 18 months (*ρ* = − 0.18; *p* = 0.022) of age, but not associated at 12 months (*ρ* = − 0.09; *p* = 0.25). Neither infant birthweight nor gestational age was associated with infant TL at any time point (ps > 0.081).

**Table 2:** Demographic characteristics and relevant covariates of study participants

Demographic outcome, mean (SD)	Total (***N*** = 181)	No prenatal smoking (***n*** = 152)	Prenatal smoking (***n*** = 29)	***p***-value
Maternal conception age (year)	28.64 (5.62)	28.38 (5.68)	29.97 (5.19)	0.17
Infant birthweight (kg)	3.26 (0.57)	3.29 (0.58)	3.11 (0.52)	0.15
SES	3.07 (1.94)	3.20 (1.92)	2.35 (1.90)	**0.028**
	% (*n*)	% (*n*)	% (*n*)	
**Race**				0.24
Black	51.9 (94)	88.3 (83)	11.7 (11)	
White	37.0 (67)	79.1 (53)	20.9 (14)	
Other	11.1 (20)	80.0 (16)	20.0 (4)	
**Baby sex**				0.98
Male	51.9 (94)	84.0 (79)	16.0 (15)	
Female	48.1 (87)	83.9 (73)	16.1 (14)	
**Pregnancy complications**				0.26
No	82.3 (149)	82.5 (123)	17.5 (26)	
Yes	17.7 (32)	90.6 (29)	9.4 (3)	
**Maternal postnatal depression (18 months)**				0.26
No	85.8 (133)	88.0 (117)	12.0 (16)	
Yes	14.2 (16)	59.1 (13)	40.9 (9)

**Table 3:** Correlation matrix of study outcomes and relevant covariates

	4-month TL	12-month TL	18-month TL	ADHD *T*-score	Prenatal smoking	Infant birthweight	Sex	Race	SES	Maternal pregnancy complications
4-month TL	1									
12-month TL	0.24**	1								
18-month TL	0.25**	0.24**	1							
ADHD *T*-score	− 0.02	− 0.05	− 0.05	1						
Prenatal smoking	0.15	− 0.12	− 0.18*	0.13	1					
Infant birthweight	− 0.04	− 0.14	0.02	0.05	− 0.17*	1				
Sex	− 0.01	− 0.05	− 0.00	− 0.12	0.00	− 0.06	1			
Race	0.02	− 0.21**	− 0.09	0.07	0.12	0.29****	0.05	1		
SES	0.02	− 0.18*	0.02	− 0.04	− 0.17*	0.30****	0.03	0.40****	1	
Pregnancy complications	− 0.05	− 0.01	− 0.02	− 0.10	− 0.08	− 0.22**	0.02	− 0.20**	− 0.09	1
Maternal postnatal depression	0.05	0.10	− 0.13	0.23**	0.26**	− 0.09	− 0.05	− 0.15	− 0.27***	0.10

Prenatal smoking: 0 = no, 1 = yes; sex: 1 = male, 2 = female; race: 0 = Black, 1 = White, 2 = other; pregnancy complications: 0 = no, 1 = yes

*p < 0.05, **p < 0.01; ***p < 0.001; ****p < 0.0001

There was a higher prevalence of maternal postnatal depression in mothers who reported prenatal smoking (*ρ* = 0.26; *p* = 0.001). Mothers reporting depression also had lower SES (*ρ* = − 0.27; *p* = 0.0005). Maternal depression was not associated with infant TL at any age (Table 3) or with the trajectory of infant TL (*β* = − 0.109, 95% CL [− 0.335, 0.116]; *p* = 0.34).

### The impact of maternal prenatal smoking on infant TL trajectory

**Fig. 2:**
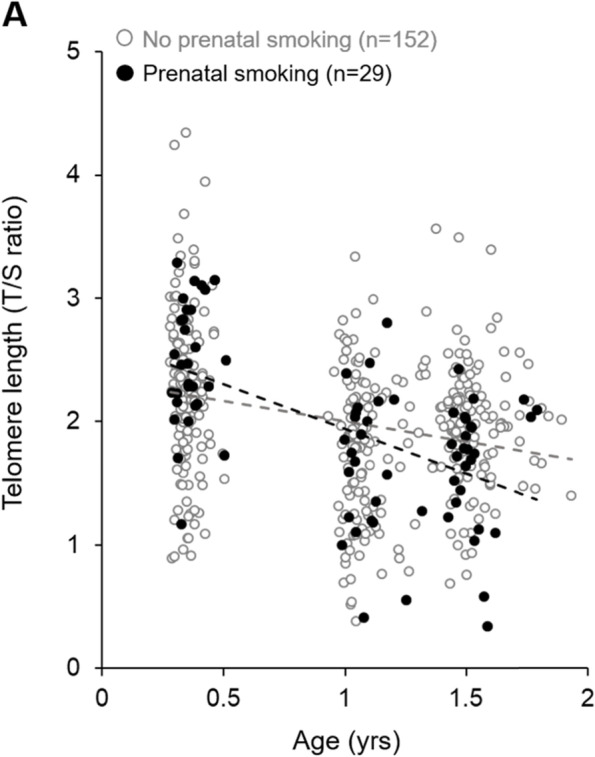
The impact of maternal prenatal smoking on the trajectory of infant telomere length. A scatter plot of infant TL across the first 18 months of age is presented. Infants whose mothers reported smoking during pregnancy are represented by black circles and infants of nonsmokers are represented by grey-outlined white circles. Linear trend lines are fitted to each group to visualize the change in TL over time

In the unadjusted model including linear age, non-linear age, and maternal prenatal smoking, prenatal smoking did not predict infant TL (Table 4; Model 1; *β* = − 0.038, 95% CL [− 0.192, 0.117]; *p* = 0.63). To examine whether maternal prenatal smoking influenced the change in TL across the first 18 months of infant age, we examined the interaction of maternal prenatal smoking and infant age. The interaction revealed a significant influence of maternal prenatal smoking on infant TL trajectory, where infants exposed to tobacco *in utero* exhibited a greater rate of TL erosion across the first 18 months of age (Fig. 2; *β* = − 0.410, 95% CL [− 0.655, − 0.166]; *p* = 0.001). In the adjusted model controlling for race, infant sex, infant birthweight, and SES, maternal prenatal smoking remained a significant predictor of greater TL attrition (Table 5; model 2; *β* = − 0.409, 95% CL [− 0.654, − 0.164]; *p* = 0.001). Maternal age at conception was included in statistical models and did not significantly contribute to these models and was therefore excluded as a covariate.

**Table 4:** The impact of maternal prenatal smoking on infant TL erosion

	Model 1: Main effects		Model 2: Adjusted model	
Sample size	181 (499 obs.)		181 (499 obs.)	
	***Β***	***p***-value	***Β***	***p***-value
**Age**	**− 1.699**	**< 0.0001**	**− 1.629**	**< 0.0001**
**Age**^**2**^	**0.715**	**< 0.0001**	**0.713**	**< 0.0001**
**Prenatal smoking**	− 0.038	0.63	**0.355**	**0.015**
**Prenatal smoking x age**			**− 0.409**	**0.001**
**Sex**			− 0.029	0.62
**Race**			− 0.053	0.26
**Birthweight**			− 0.009	0.86
**SES**			− 0.013	0.46

**Table 5:** The impact of maternal prenatal smoking on infant ADHD *T*-score and the influence of TL attrition

	Model 1: Main effects		Model 2: Adjusted model		Model 3: Adjusted model with TL attrition		Model 4: Moderation	
Sample size	133		133		133		133	
	***β***	***p***-value	***Β***	***p***-value	***β***	***p***-value	***β***	***p***-value
**Intercept**	**53.735**	**< 0.0001**	**54.934**	**< 0.0001**	**55.048**	**< 0.0001**	**54.663**	**< 0.0001**
**Prenatal smoking**	**3.453**	**0.017**	**3.706**	**0.016**	**4.045**	**0.013**	**9.410**	**0.0004**
**TL attrition**					− 0.432	0.53	0.114	0.87
**Prenatal smoking x TL attrition**							**− 5.797**	**0.010**
**Sex**			− 0.651	0.49	− 0.660	0.49	− 0.702	0.45
**Race**			− 0.394	0.59	− 0.472	0.52	− 0.533	0.46
**SES**			− 0.000	0.99	0.023	0.93	0.115	0.66

Given the association between maternal prenatal smoking and maternal depression, we stratified analyses by maternal depression. In non-depressed mothers, maternal prenatal smoking significantly predicted greater TL attrition (*β* = − 0.543, 95% CL [− 0.878, − 0.209]; *p* = 0.0016); however, no association was found in depressed mothers (*β* = − 0.196, 95% CL [− 0.573, 0.181]; *p* = 0.30).

### The impact of maternal prenatal smoking on ADHD symptoms

For all infants, ADHD subscale *T*-score was 54.15 ± 5.47 (SD). Thirty-five infants (22.6%) had ADHD *T*-scores above 60, and of these infants, *n* = 11 (7.1%) had ADHD *T*-scores equal to or greater than 65. Of these 35 infants, *n* = 9 (25.7%) had mothers who reported prenatal smoking. In infants whose mothers reported prenatal smoking, the mean ADHD *T*-score was 57.19 ± 7.91, while for infants whose mothers did not report prenatal smoking, the mean ADHD *T*-score was 53.74 ± 4.95. Maternal depression at 18 months of age was associated with ADHD *T*-score (*β* = 4.713, 95% CI [2.073, 7.354]; *p* = 0.0006) and maternal prenatal smoking predicted maternal depression at 18 months of infant age (odds ratio (OR): 4.614, 95% CI [1.733, 12.282]; *p* = 0.002). As such, post hoc analyses of maternal prenatal smoking and infant ADHD *T*-score at 18 months of age was conducted only in non-depressed mothers (*n* = 133). In non-depressed mothers, *n* = 23 infants (17.9%) had ADHD *T*-scores above 60, and seven infants (5.3%) had ADHD *T*-scores equal to or greater than 65. Within non-depressed mothers, prenatal smoking was associated with higher ADHD *T*-score (Table 5; model 1; *β* = 3.452, 95% CI [0.620, 6.285]; *p* = 0.017). After adjusting for infant sex, race, and SES, the association remained significant (Table 5; model 2; *β* = 3.706, 95% CI [0.713, 6.699]; *p* = 0.016).

### The impact of TL attrition on the relation between prenatal smoking and ADHD symptoms

**Fig. 3:**
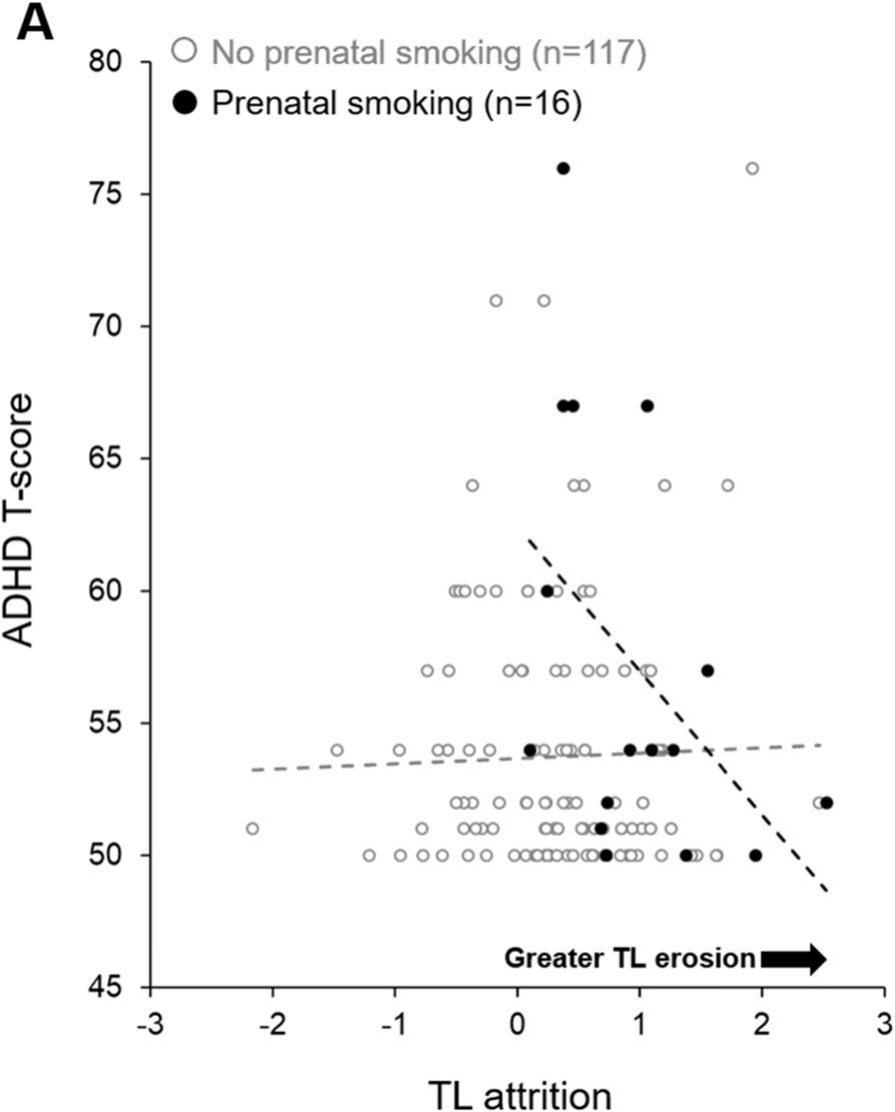
The moderation of maternal prenatal smoking and infant ADHD T-score by TL attrition. A scatter plot of infant TL attrition from 4 to 18 months of life (i.e., ∆TL = 4-month TL–18- month TL) against ADHD *T*-score is presented by maternal prenatal smoking groups. Infants whose mothers reported smoking during pregnancy are represented by black circles and infants of nonsmokers are represented by grey-outlined white circles. Linear trend lines are fitted to each group to visualize the relation between TL attrition and ADHD T-score

As neither TL nor TL attrition were associated with infant ADHD *T*-score, moderation was tested. Moderation analyses, conducted only in non-depressed mothers, utilized the same covariate structure as the independent association of maternal prenatal smoking and infant ADHD *T*-score and included sex, race, and SES. The relation between maternal prenatal smoking and ADHD *T*-score was moderated by TL attrition (Table 5; model 4; *β* = − 5.797, 95% CI [− 10.207, − 1.386]; *p* = 0.010). Visualization of the interaction in Fig. 3 indicates that, in infants of mothers who reported prenatal smoking, *higher* ADHD *T*-scores were associated with less TL erosion from four to 18 months of age. Maternal postnatal smoking was also considered; however, despite the positive correlation between maternal prenatal smoking and maternal postnatal smoking, maternal postnatal smoking itself was not associated with infant TL at any time point nor with ADHD symptoms.

## Discussion

Maternal report of prenatal smoking was significantly associated with infant TL attrition across the first 18 months of life. Even after accounting for SES, race, birth weight, and infant sex, maternal prenatal smoking was associated with a greater rate of TL shortening from four to 18 months of infant age. These results are consistent with cross-sectional studies linking maternal prenatal smoking to shorter offspring TL and augment previous findings linking maternal prenatal smoking, secondhand smoke exposure, and maternal cadmium levels with alterations in offspring TL both in newborns and across childhood [20, 21, 23–27]. Further, our findings provide initial evidence that altered TL trajectories, as a function of prenatal maternal smoking, is one mechanistic pathway that contributes to the lasting negative health effects of maternal smoking for health outcomes in offspring [43].

Consistent with meta-analytic findings, in this relatively small cohort, prenatal smoking was correlated with maternal report of ADHD symptoms in her 18-month-old child. In this cohort, a significant association was found between maternal report of depressive symptoms and both prenatal smoking and ADHD symptoms in her toddler at 18 months. As our modest sample size precluded testing of mediation, larger studies with adequate power may be useful in determining this relationship.

As our primary initial hypothesis was to examine TL as a biological pathway linking maternal prenatal smoking and offspring ADHD, we subsequently report that TL change across the first 18 months of life moderated the association between prenatal smoking and early symptoms of ADHD in non-depressed mothers. For children whose mothers reported prenatal smoking, higher maternal report of ADHD symptoms at 18 months of age was associated with *lower* TL attrition. While initially counter-intuitive, these findings fit with existing neurodevelopmental models of ADHD that suggest delayed cortical maturation contributes to ADHD symptoms and also with previous reports of longer TL in subpopulations of cord blood lymphocytes with maternal prenatal smoking [26]. Almanzar et al. suggest that maternal prenatal smoking results in the preferential loss of older lymphocytes, resulting in a younger population of circulating cells with longer TL. Alternatively, similar to previous studies that correlated telomere biology with oligodendrocyte maturation, one proposed hypothesis is that the delayed maturation and differentiation of the neurons in the prefrontal cortex associated with ADHD may be associated with longer TL [44]. If TL is reflective of cellular replicative history [26, 44], the finding of less TL attrition may be reflective of slower overall neurodevelopmental processes reflecting decreased cellular differentiation and less mature cell populations. Notably, as this study reports on TL measured from buccal epithelial cells, which are neuroectodermally derived, while previous studies have utilized mesodermally derived lymphocytes, the relation between neurodevelopmental trajectories and buccal TL may be more tightly aligned. Most recently, Cicek et al. reported an association between telomerase, the enzyme that elongates telomeres, and both ADHD symptoms and cognitive function associated with ADHD. The authors hypothesized that neuroinflammation, one mechanism increasingly tied to ADHD, resulted in elevated telomerase, which, while not measured in this study, would theoretically predict longer telomere length [45]. While highly speculative, the moderation by longer TL may reflect the influence of smoking induced inflammation triggering elevated telomerase and ADHD, perhaps indicating that while smoking contributes to this pathway the effect on TL is through telomerase and the relation between smoking and ADHD may be driven by inflammation, as such our findings reflect joint consequences of inflammation rather than more standard considerations of elevated oxidative stress and subsequent shorter TL. Future studies targeting additional markers of cellular differentiation, including telomerase and cellular markers of DNA damage, as well as explorations of the relation with inflammation, complimented by preclinical animal models, are needed to better disentangle these complex pathways.

While these results supplement the growing body of literature relating early childhood TL and prenatal tobacco exposure, they are not without limitations. Despite the repeated TL measurements within infants, this remains a modest sample in which to examine TL as both a marker of exposure and a predictor of a health outcome. Particularly given concerns about assay repeatability with the qPCR-based TL measurement, close attention to both sample size and assay repeatability calculated with ICCs is necessary. The calculated ICCs for the TL assays in this cohort were high, and, using available sample size estimates (tulane.trn.edu) and the calculated ICC > 0.90, we detected significant associations between the change in TL and prenatal smoking [41]. Another limitation is the reliance on maternal self-report and medical record data for the determination of maternal prenatal smoking [41, 46]. Our measure of exposure is limited to maternal use and did not capture additional sources of tobacco exposure, such as paternal smoking or maternal second-hand smoke exposure, nor were additional exposures such as alcohol, marijuana, prescribed or illicit drug use, or environmental contaminants, which are often co-occurring with tobacco use, included in our study design. Our data did not permit exploration of testing a dose-response effect of smoke exposure on TL. Additionally, our small sample size prohibited consideration of additional pregnancy covariates (i.e. pre-eclampsia). As there is sparse evidence to suggest a directionality of the relationship between TL attrition and ADHD symptomatology during infancy, challenges exist in discussing potential molecular mechanisms. Furthermore, the focus of this study is on prenatal exposure and did not capture postnatal smoking exposure; however, large-scale studies demonstrate that prenatal exposure is more predictive of negative health outcomes than postnatal exposure [47, 48]. Although our primary aim was to examine the impact of prenatal smoking, we subsequently examined if maternal postnatal smoking contributed to additional variance in the model. Maternal postnatal smoking was collected via self-report when the infant was 4 and 12 months of age. Although maternal prenatal smoking was positively correlated with maternal postnatal smoking, maternal postnatal smoking was not associated with infant TL at any time nor with ADHD symptoms. Since meta-analyses evaluating telomere attrition rates in adult smokers do not demonstrate greater attrition in smokers compared to non-smokers [17], further studies are needed to evaluate TL attrition at various life stages to help direct clinicians to the most influential point of exposure as it relates to long-term outcomes. As maternal depression has been associated with associated with both higher risk of maternal prenatal smoking [49, 50] and early childhood ADHD symptoms [51], depressed mothers were excluded from our moderation analysis; future studies sufficiently powered to test the biological pathways in women with depression are needed. Given the potential role of prematurity in infant TL dynamics and ADHD symptoms as a function of maternal prenatal smoking, the analyses excluding infants with gestational ages less than 37 weeks we run and the findings were consistent. We did not consider shared genetic factors that would be related to smoking behaviors and TL [52], and although there is evidence of heritability in telomere length to date there is no evidence of shared genetic risk with smoking behaviors and telomere length [53], future studies accounting for genetic contributions are warranted. Finally, maternal report of symptoms was done at a very early age and was based on the CBCL subscale scores, which are not diagnostic of ADHD, as such caution is warranted. However, the correlation between ADHD subscale scores and later diagnosis of ADHD is high although later variability is possible. Additional studies are needed to better understand the relation between prenatal exposure to smoking, the trajectory of telomere length attrition and socioemotional and behavioral outcomes throughout childhood.

## Conclusions

Our findings confirm previously published studies linking offspring TL and prenatal maternal smoking and support the literature base demonstrating the intergenerational consequences of prenatal tobacco exposure. Previous data reiterates that continued smoking during pregnancy or third trimester smoking is linked to worse outcomes than for those mothers who either never smoked or stopped smoking early in pregnancy [4, 54]. In the future, TL may serve as a predictive index of later offspring health risks. Our findings provide a foundation to investigate this further from both a clinical and a mechanistic standpoint. Detailed prospective studies evaluating the trajectory of TL in children of mothers who smoke prenatally, from birth through adulthood, and the correlation with long-term negative health outcomes are needed to better understand these pathways. In addition, larger studies adequate powered to explore dose and duration of exposure may define sensitive periods of heightened neurodevelopment risk and additional factors in both the prenatal and the postnatal environment (i.e. gestational diabetes, pre-eclampsia) that can moderate long-term outcomes.

Given the accumulating public health concerns related to multiple prenatal substance exposures, evaluation of the independent and interactive effect of multiple substances of abuse (e.g., marijuana, opiates) on TL and long-term infant outcomes is an important future research direction. Despite well-documented adverse consequences of prenatal smoking, it remains unfortunately prevalent. To better understand the pathophysiologic mechanisms by which tobacco smoking during pregnancy influences the next generation, multigenerational studies could shed light on critical events and outcomes during and after fetal development.

## Supplementary Information

**Additional file 1: Figure S1.** The impact of maternal prenatal smoking on the trajectory of infant telomere. A scatter plot of infant TL across the first 18 months of age is presented by maternal prenatal smoking status for all infants (A) and for infants whose mothers did not report postnatal depression when infants were 18-months of age (B). Panel B presents the sub-sample of infants included in the moderation analysis of maternal prenatal smoking on infant ADHD symptoms by infant TL attrition (i.e., study 2). Infants whose mothers smoked during pregnancy are represented by black circles and infants of nonsmokers are represented by grey outlined white circles. Linear trend lines are fitted to each group to visualize the change in TL over time.

## Data Availability

The datasets generated and/or analyzed during the current study are not publicly available but are available from the corresponding author on reasonable request.

## Abbreviations

ADHD: Attention deficit/hyperactivity disorder
CBCL: Child Behavior Check List
CI: Confidence interval
CL: Confidence limits
CV: Coefficients of variations
MLM: Multilevel mixed-effects linear regression models
OR: Odds ratio
PCR: Polymerase chain reaction
SES: Socioeconomic status
TL: Telomere length
WIC: Women, Infants and Children

## References

[1] Drake P, Driscoll AK, Mathews TJ. Cigarette smoking during pregnancy: United States, 2016. NCHS Data Brief. 2018;305:1–8. PMID: 29528282. 29528282

[2] McCowan LM, Dekker GA, Chan E, Stewart A, Chappell LC, Hunter M, Moss-Morris R, North RA; SCOPE consortium. Spontaneous preterm birth and small for gestational age infants in women who stop smoking early in pregnancy: prospective cohort study. BMJ. 2009;338:b1081. 10.1136/bmj.b1081. Erratum in: BMJ. 2009;338. 10.1136/bmj.b1558. 10.1136/bmj.b1081 PMC2661373 19325177

[3] Salihu HM, Wilson RE. Epidemiology of prenatal smoking and perinatal outcomes. Early Hum Dev. 2007;83(11):713-20. 10.1016/j.earlhumdev.2007.08.002. 10.1016/j.earlhumdev.2007.08.002 17884310

[4] Bernstein IM, Mongeon JA, Badger GJ, Solomon L, Heil SH, Higgins ST. Maternal smoking and its association with birth weight. Obstet Gynecol. 2005;106(5 Pt 1):986-91. 10.1097/01.AOG.0000182580.78402.d2. 10.1097/01.AOG.0000182580.78402.d2 16260516

[5] Burke H, Leonardi-Bee J, Hashim A, Pine-Abata H, Chen Y, Cook DG (2012). Prenatal and passive smoke exposure and incidence of asthma and wheeze: systematic review and meta-analysis. Pediatrics..

[6] Rayfield S, Plugge E. Systematic review and meta-analysis of the association between maternal smoking in pregnancy and childhood overweight and obesity. J Epidemiol Community Health. 2017;71(2):162-173. 10.1136/jech-2016-207376. 10.1136/jech-2016-207376 27480843

[7] Durmuş B, Heppe DH, Taal HR, Manniesing R, Raat H, Hofman A, Steegers EA, Gaillard R, Jaddoe VW. Parental smoking during pregnancy and total and abdominal fat distribution in school-age children: the Generation R Study. Int J Obes (Lond). 2014;38(7):966-72. 10.1038/ijo.2014.9. 10.1038/ijo.2014.9 24448598

[8] Dong T, Hu W, Zhou X, Lin H, Lan L, Hang B (2018). Prenatal exposure to maternal smoking during pregnancy and attention-deficit/hyperactivity disorder in offspring: A meta-analysis. Reprod Toxicol.

[9] Huang L, Wang Y, Zhang L, Zheng Z, Zhu T, Qu Y (2018). Maternal smoking and attention-deficit/hyperactivity disorder in offspring: a meta-analysis. Pediatrics..

[10] Makadia LD, Roper PJ, Andrews JO, Tingen MS (2017). Tobacco Use and smoke exposure in children: new trends, harm, and strategies to improve health outcomes. Curr Allergy Asthma Rep.

[11] Health CfDCaPNCfCDPaHPOoSa (2010). How tobacco smoke causes disease: the biology and behavioral basis for smoking-attributable disease: a report of the surgeon general.

[12] Garrabou G, Hernandez AS, Catalan Garcia M, Moren C, Tobias E, Cordoba S (2016). Molecular basis of reduced birth weight in smoking pregnant women: mitochondrial dysfunction and apoptosis. Addict Biol.

[13] Astuti Y, Wardhana A, Watkins J, Wulaningsih W (2017). Cigarette smoking and telomere length: a systematic review of 84 studies and meta-analysis. Environ Res.

[14] Savage SA, Bertuch AA (2010). The genetics and clinical manifestations of telomere biology disorders. Genet Med.

[15] Valdes AM, Andrew T, Gardner JP, Kimura M, Oelsner E, Cherkas LF (2005). Obesity, cigarette smoking, and telomere length in women. Lancet..

[16] Khan RJ, Gebreab SY, Gaye A, Crespo PR, Xu R, Davis SK (2019). Associations of smoking indicators and cotinine levels with telomere length: National Health and Nutrition Examination Survey. Prev Med Rep.

[17] Bateson M, Aviv A, Bendix L, Benetos A, Ben-Shlomo Y, Bojesen SE (2019). Smoking does not accelerate leucocyte telomere attrition: a meta-analysis of 18 longitudinal cohorts. R Soc Open Sci.

[18] Factor-Litvak P, Susser E, Kezios K, McKeague I, Kark JD, Hoffman M, et al. Leukocyte telomere length in newborns: implications for the role of telomeres in human disease. Pediatrics. 2016;137(4). 10.1542/peds.2015-3927 PMC4811318 26969272

[19] Benetos A, Kark JD, Susser E, Kimura M, Sinnreich R, Chen W (2013). Tracking and fixed ranking of leukocyte telomere length across the adult life course. Aging Cell.

[20] Ip P, Chung BHY, Ho FKW, Chan GCF, Deng W, Wong WHS (2016). Prenatal tobacco exposure shortens telomere length in children. Nicotine Tob Res.

[21] Liu B, Song L, Zhang L, Wu M, Wang L, Cao Z, et al. Prenatal second-hand smoke exposure and newborn telomere length. Pediatr Res. 2019. 10.1038/s41390-019-0594-2 31578036

[22] Osorio-Yáñez C, Clemente DBP, Maitre L, Vives-Usano M, Bustamante M, Martinez D, et al. Early life tobacco exposure and children's telomere length: the HELIX project. (1879-1026 (Electronic)). 10.1016/j.scitotenv.2019.135028 32000334

[23] Mirzakhani H, De Vivo I, Leeder JS, Gaedigk R, Vyhlidal CA, Weiss ST (2017). Early pregnancy intrauterine fetal exposure to maternal smoking and impact on fetal telomere length. Eur J Obstet Gynecol Reprod Biol.

[24] Salihu HM, Pradhan A, King L, Paothong A, Nwoga C, Marty PJ (2015). Impact of intrauterine tobacco exposure on fetal telomere length. Am J Obstet Gynecol.

[25] Theall KP, McKasson S, Mabile E, Dunaway LF, Drury SS (2013). Early hits and long-term consequences: tracking the lasting impact of prenatal smoke exposure on telomere length in children. Am J Public Health.

[26] Almanzar G, Eberle G, Lassacher A, Specht C, Koppelstaetter C, Heinz-Erian P (2013). Maternal cigarette smoking and its effect on neonatal lymphocyte subpopulations and replication. BMC Pediatr.

[27] Zhang L, Song L, Liu B, Wu M, Wang L, Zhang B, et al. Prenatal cadmium exposure is associated with shorter leukocyte telomere length in Chinese newborns. 10.1186/s12916-019-1262-4 PMC6364384 30722777

[28] Yeh JK, Wang CY. Telomeres and telomerase in cardiovascular diseases. Genes (Basel). 2016;7(9). 10.3390/genes7090058 PMC5042389 27598203

[29] Clemente DBP, Maitre L, Bustamante M, Chatzi L, Roumeliotaki T, Fossati S (2019). Obesity is associated with shorter telomeres in 8 year-old children. Sci Rep.

[30] Wang J, Dong X, Cao L, Sun Y, Qiu Y, Zhang Y, et al. Association between telomere length and diabetes mellitus: a meta-analysis. J Int Med Res. 2016;44. 10.1177/0300060516667132 PMC5536737 28322101

[31] Barnes RP, Fouquerel E, Opresko PL (2019). The impact of oxidative DNA damage and stress on telomere homeostasis. Mech Ageing Dev.

[32] Di Micco R, Krizhanovsky V, Baker D, d'Adda di Fagagna F (2021). Cellular senescence in ageing: from mechanisms to therapeutic opportunities. Nat Rev Mol Cell Biol.

[33] Costa Dde S, Rosa DV, Barros AG, Romano-Silva MA, Malloy-Diniz LF, Mattos P (2015). Telomere length is highly inherited and associated with hyperactivity-impulsivity in children with attention deficit/hyperactivity disorder. Front Mol Neurosci.

[34] Momany AM, Lussier S, Nikolas MA, Stevens H. Telomere length and ADHD symptoms in young adults. J Atten Disord. 2019;1087054719865776. 10.1177/1087054719865776 PMC7202341 31370740

[35] Krieger N, Chen Jt, Waterman PD, Soobader MJ, Subramanian SV, Carson R. Choosing area based socioeconomic measures to monitor social inequalities in low birth weight and childhood lead poisoning: The Public Health Disparities Geocoding Project (US). (0143-005X (Print)). 10.1136/jech.57.3.186 PMC1732402 12594195

[36] Jones CW, Gambala C, Esteves KC, Wallace M, Schlesinger R, O’Quinn M (2017). Differences in placental telomere length suggest a link between racial disparities in birth outcomes and cellular aging. Am J Obstet Gynecol.

[37] Beck AT, Steer RA, Brown GK. Beck depression inventory-II. San Antonio; 1996. p. 78204–2498.

[38] Achenbach TM, Edelbrock CS (1983). Manual for the child behavior checklist: and revised child behavior profile.

[39] Cawthon RM (2009). Telomere length measurement by a novel monochrome multiplex quantitative PCR method. Nucleic Acids Res.

[40] Esteves KC, Jones CW, Wade M, Callerame K, Smith AK, Theall KP (2020). Adverse childhood experiences: implications for offspring telomere length and psychopathology. Am J Psychiatry.

[41] Lindrose AR, McLester-Davis LWY, Tristano RI, Kataria L, Gadalla SM, Eisenberg DTA, et al. Method comparison studies of telomere length measurement using qPCR approaches: a critical appraisal of the literature. bioRxiv. 2020:2020.09.04.282632. 10.1371/journal.pone.0245582 PMC7817045 33471860

[42] Graham JW (2009). Missing data analysis: making it work in the real world. Annu Rev Psychol.

[43] Whiteman VE, Goswami A, Salihu HM. Telomere length and fetal programming: a review of recent scientific advances. Am J Reprod Immunol (New York, NY : 1989). 2017;77(5). 10.1111/aji.12661 28500672

[44] Caporaso GL, Chao MV (2001). Telomerase and oligodendrocyte differentiation. J Neurobiol.

[45] Uzun Cicek AA-O, Mercan Isik CA-O, Bakir SA-O, Ulger DA-OX, Sari SA-O, Bakir DA-O, et al. Evidence supporting the role of telomerase, MMP-9, and SIRT1 in attention-deficit/hyperactivity disorder (ADHD). (1435-1463 (Electronic)). 10.1007/s00702-020-02231-w 32691156

[46] Eisenberg DTA (2016). Telomere length measurement validity: the coefficient of variation is invalid and cannot be used to compare quantitative polymerase chain reaction and Southern blot telomere length measurement techniques. Int J Epidemiol.

[47] McEvoy CT, Spindel ER (2017). Pulmonary effects of maternal smoking on the fetus and child: effects on lung development, respiratory morbidities, and life long lung health. Paediatr Respir Rev.

[48] Neuman A, Hohmann C, Orsini N, Pershagen G, Eller E, Kjaer HF (2012). Maternal smoking in pregnancy and asthma in preschool children: a pooled analysis of eight birth cohorts. Am J Respir Crit Care Med.

[49] Cui M, Kimura T, Ikehara S, Dong JY, Ueda K, Kawanishi Y, et al. Prenatal tobacco smoking is associated with postpartum depression in Japanese pregnant women: the Japan Environment and Children’s Study. (1573-2517 (Electronic)). 10.1016/j.jad.2019.11.145 31846904

[50] Smedberg J, Lupattelli A, Mårdby A-C, Øverland S, Nordeng H (2015). The relationship between maternal depression and smoking cessation during pregnancy—a cross-sectional study of pregnant women from 15 European countries. Arch Womens Ment Health.

[51] Cheung K, Aberdeen K, Ward MA, Theule J (2018). Maternal depression in families of children with ADHD: a meta-analysis. J Child Fam Stud.

[52] Loukola A, Hällfors J, Korhonen T, Kaprio J (2014). Genetics and smoking. Curr Addict Rep.

[53] Broer L, Codd V, Nyholt DR, Deelen J, Mangino M, Willemsen G (2013). Meta-analysis of telomere length in 19,713 subjects reveals high heritability, stronger maternal inheritance and a paternal age effect. Eur J Hum Genet.

[54] Jaddoe VW, Troe EJW, Hofman A, Mackenbach JP, Moll HA, Steegers EA (2008). Active and passive maternal smoking during pregnancy and the risks of low birthweight and preterm birth: the Generation R Study. Paediatr Perinat Epidemiol.

