# Supplementary material for: Impact of prenatal tobacco smoking on infant telomere length trajectory and ADHD symptoms at 18 months: a longitudinal cohort study
Source: BMC Med. 2022 Apr 28;20:153. doi: 10.1186/s12916-022-02340-1 (PMC9047258; doi:10.1186/s12916-022-02340-1)
Supplement: Supplementary file 1 — Additional file 1: Figure S1. The impact of maternal prenatal smoking on the trajectory of infant telomere. A scatter plot of infant TL across the first 18 months of age is presented by maternal prenatal smoking status for all infants (A) and for infants whose mothers did not report postnatal depression when infants were 18-months of age (B). Panel B presents the sub-sample of infants included in the moderation analysis of maternal prenatal smoking on infant ADHD symptoms by infant TL attrition (i.e., study 2). Infants whose mothers smoked during pregnancy are represented by black circles and infants of nonsmokers are represented by grey outlined white circles. Linear trend lines are fitted to each group to visualize the change in TL over time. [file 12916_2022_2340_MOESM1_ESM.docx]

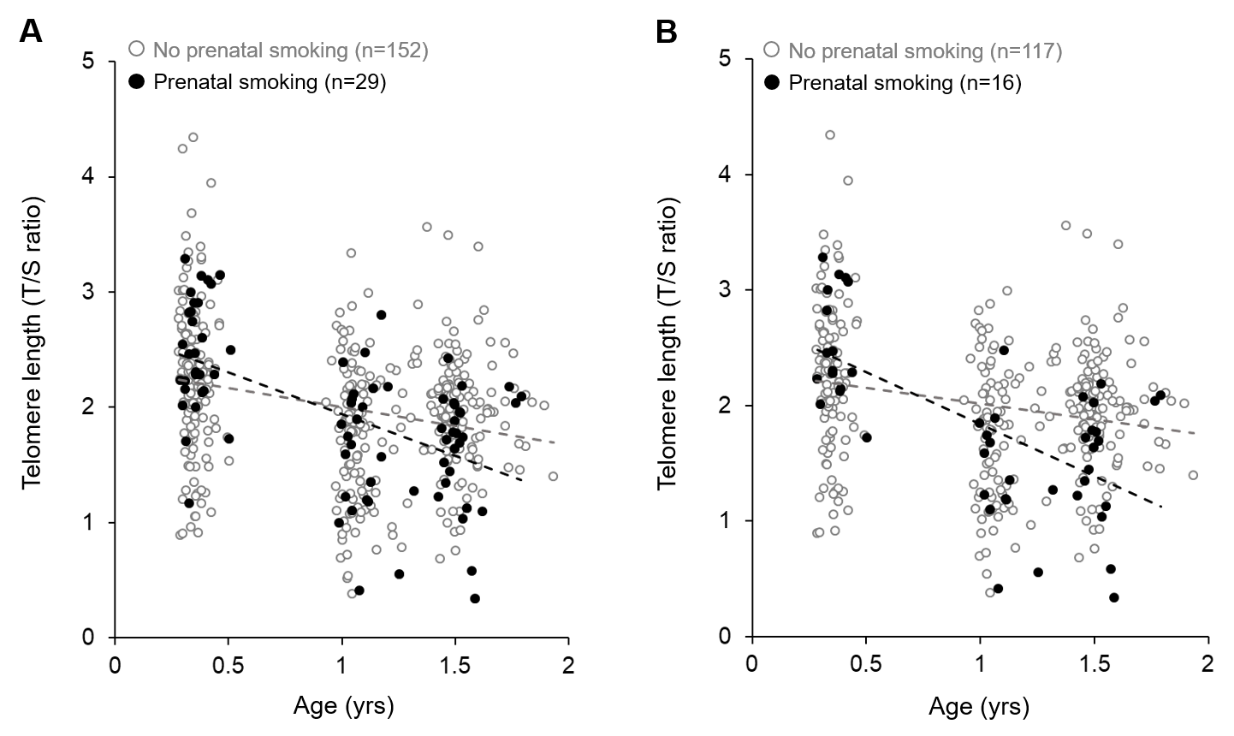


***Figure S1. The impact of maternal prenatal smoking on the trajectory of infant telomere.*** *A scatter plot of infant TL across the first 18 months of age is presented by maternal prenatal smoking status for all infants (A) and for infants whose mothers did not report postnatal depression when infants were 18-months of age (B). Panel B presents the sub-sample of infants included in the moderation analysis of maternal prenatal smoking on infant ADHD symptoms by infant TL attrition (i.e., study 2). Infants whose mothers smoked during pregnancy are represented by black circles and infants of nonsmokers are represented by grey outlined white circles. Linear trend lines are fitted to each group to visualize the change in TL over time.*
